# Atypical Presentation of Disseminated Tuberculosis With Third Cranial Nerve Palsy and Aortic Aneurysm: A Case Report

**DOI:** 10.7759/cureus.92517

**Published:** 2025-09-17

**Authors:** Arundhathy Krishna, Krishna S Pavuluri

**Affiliations:** 1 Cardiology, Surrey and Sussex Healthcare NHS Trust, Redhill, GBR; 2 General Internal Medicine, Sri Ramachandra Institute of Higher Education and Research, Chennai, IND

**Keywords:** antituberculosis therapy, diagnostic and therapeutic challenges, infectious diseases, isolated third cranial nerve palsy, sputum bronchoalveolar lavage, striato capsular infarct, tb granulomas, thoracic aortic aneurysm (taa), tree in bud appearance, tuberculous vasculopathy

## Abstract

Tuberculosis (TB) remains a leading cause of mortality among infectious diseases globally, posing significant diagnostic and therapeutic challenges despite being curable.This case highlights a delayed diagnosis of miliary TB with rare and severe complications in a patient with an atypical presentation. A 44-year-old female of African origin presented with non-specific symptoms, including fever, malaise, and deranged liver function tests, after travel to Nairobi. After nearly two months of intermittent symptoms, she developed neurological signs, including a third cranial nerve palsy, eye pain, and ptosis, prompting further investigation. Brain imaging revealed multiple enhancing nodules consistent with TB granulomas and a striato-capsular infarct. Subsequently, thoracic imaging showed a thoracic aortic aneurysm, extensive lung miliary nodularity, and “tree-in-bud” appearance, indicative of miliary tuberculosis. The patient developed tuberculous vasculopathy, likely contributing to the acute brain infarct, and a rare but life-threatening tuberculous aortic aneurysm. While cranial nerve palsies are known complications of central nervous system TB, isolated third nerve involvement and the co-occurrence of a large-vessel aneurysm are uncommon. This case emphasizes the need for a high index of suspicion for TB in patients with unusual or multifocal symptoms, even in the absence of typical signs. Early diagnosis, followed by prompt initiation of antituberculosis therapy with appropriate surgical intervention for complications such as aneurysms, is important in reducing morbidity and preventing fatalities in such complex presentations.

## Introduction

Tuberculosis (TB) remains a leading cause of mortality among infectious diseases globally, particularly in its disseminated or miliary form, which is notoriously difficult to diagnose in the absence of classic pulmonary symptoms such as chronic cough or hemoptysis [1]. Central nervous system (CNS) dissemination may present with fever, malaise, and deranged liver tests, non‑specific signs that often delay accurate diagnosis [2,3]. Miliary TB frequently mimics tropical or autoimmune pathologies, particularly in patients with recent travel to endemic regions, necessitating a broad differential diagnosis from the outset [3].

Cranial nerve palsies are recognized complications of tuberculous meningitis (TBM), with the oculomotor (III) and abducens (VI) nerves most frequently involved. Oculomotor nerve involvement occurs in approximately 52% of cranial nerve palsy cases in TBM, though isolated third nerve palsy is rare [4]. Cerebral vasculopathy and infarcts due to TBM result from inflammatory exudates in basal cisterns, leading to vasculitis, vessel narrowing, thrombosis, and vasospasm-induced strokes [4,5].

Tuberculous aortic aneurysms are exceedingly rare, with fewer than 100 cases reported in the English-language literature by 1999. These aneurysms typically arise through direct spread from adjacent infected tissues or hematogenous dissemination and are often pseudoaneurysms with high mortality if untreated [6,7]. Successful outcomes depend on prompt recognition, antituberculosis treatment, and timely surgical or endovascular repair [6].

## Case presentation

A 44-year-old woman of African origin residing in the United Kingdom, generally fit and well with a history of hypertension, initially presented with fever and malaise in February 2024. She had travelled to Nairobi four months prior. She was admitted due to deranged liver function tests (LFTs). A liver ultrasound showed features consistent with acute cholecystitis. After a week of improvement, her LFTs returned toward the normal levels, and she was discharged.

At a follow-up appointment two weeks later, laboratory results, including full blood count, urea and electrolytes, bone profile, and viral screen, were all normal or negative. A spotted rickettsial fever IgG was positive, but as she was asymptomatic, this was deemed a false-positive result. She was again discharged.

**Figure 1:**
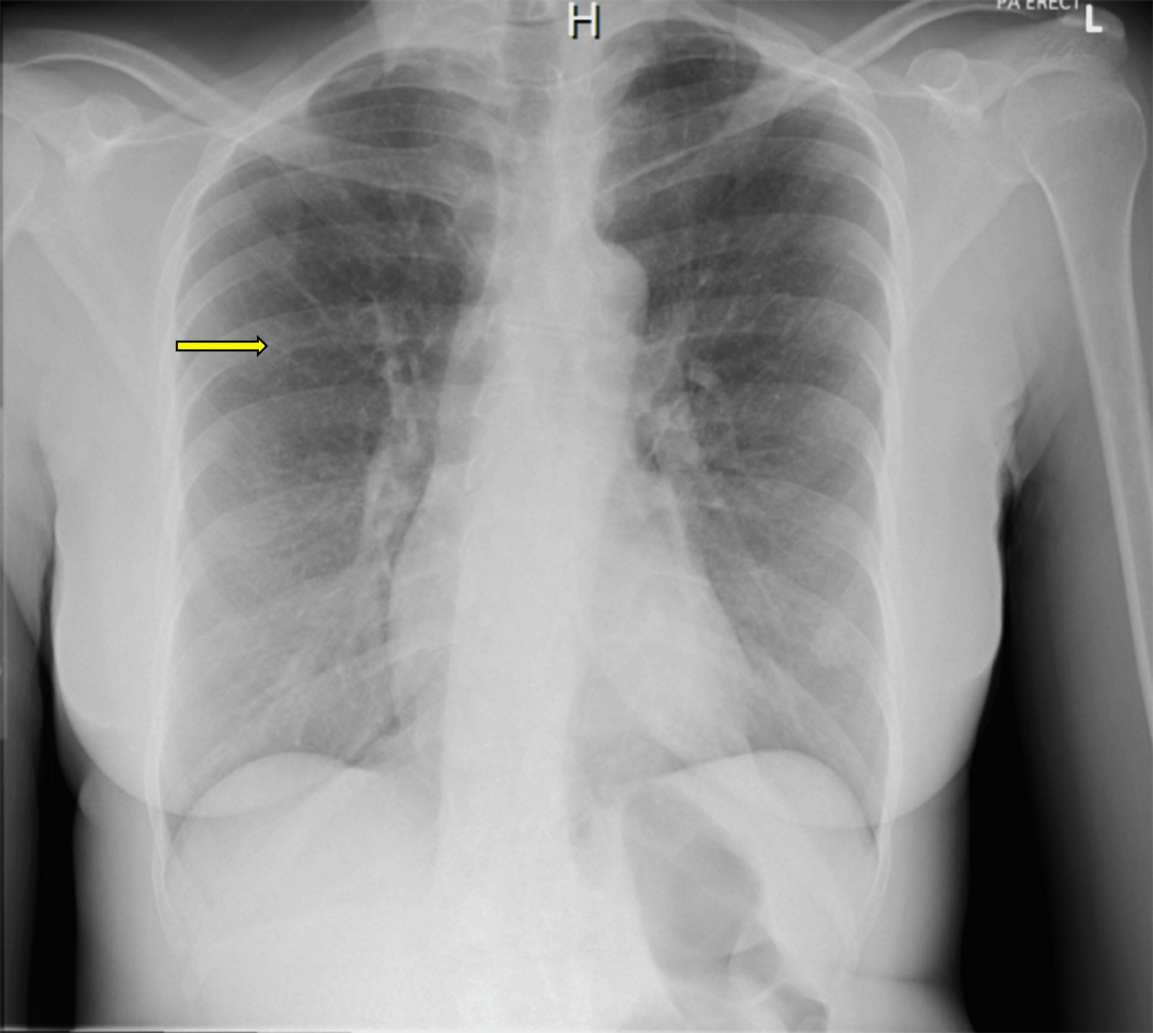
Chest X-ray showing miliary nodules and a tree-in-bud appearance (arrow).

**Figure 2:**
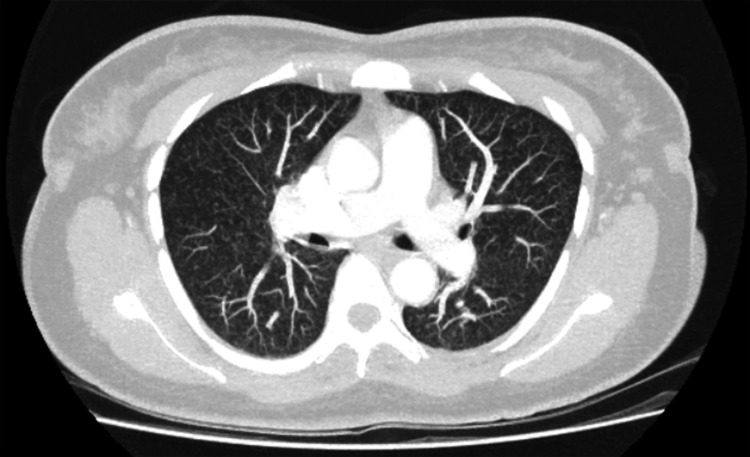
CT of the chest showing extensive nodularity throughout both lungs, suggestive of miliary tuberculosis.

One week later, she returned with a headache and fever, but was diagnosed with a tension headache and discharged. A few days later, with further worsening of symptoms, she underwent a CT of the head and lumbar puncture (LP). No intracranial bleed was detected on CT. LP showed the following findings (laboratory values with typical reference ranges): total leukocyte count, 72 × 10^6^; lymphocytes, 64% (normal range = ~40-80%); polymorphs, 36% (normal range = 0-6%); cerebrospinal fluid (CSF) protein, 1.16 g/L (normal range = 0.15-0.45); and CSF glucose, 1.8 mmol/L (normal range = 2.5-4.4 mmol/L). A chest X-ray revealed miliary nodules with a “tree-in-bud” appearance (Figure 1), and a CT of the chest confirmed widespread miliary nodules (Figure 2). Sputum and bronchoalveolar lavage polymerase chain reaction tested positive for *Mycobacterium tuberculosis*, prompting initiation of antituberculous therapy (rifampicin, pyrazinamide, ethambutol, and moxifloxacin for a year for CNS involvement) and discharge with follow-up by the TB team.

**Figure 3:**
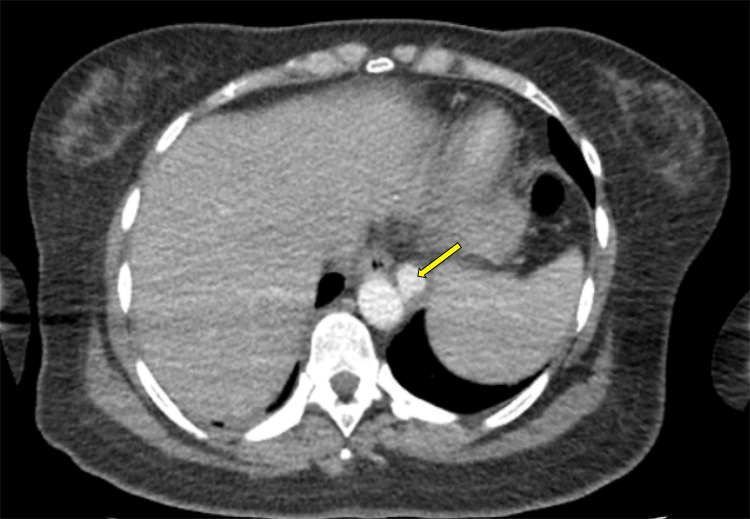
CT of the thorax showing an aneurysm in the distal thoracic aorta (arrow).

Approximately a month later, in May, she developed eye pain and ptosis and was seen in the eye casualty department. An urgent MRI of the brain was recommended within a week for suspected third cranial nerve palsy. However, she traveled to Turkey to attend a wedding, where she was admitted due to worsening headache, diplopia, restricted eye movements, and left eyelid drooping. There, she was treated for meningitis, an acute brain infarct was diagnosed, and a thoracic aortic aneurysm was discovered (Figure 3).

**Figure 4:**
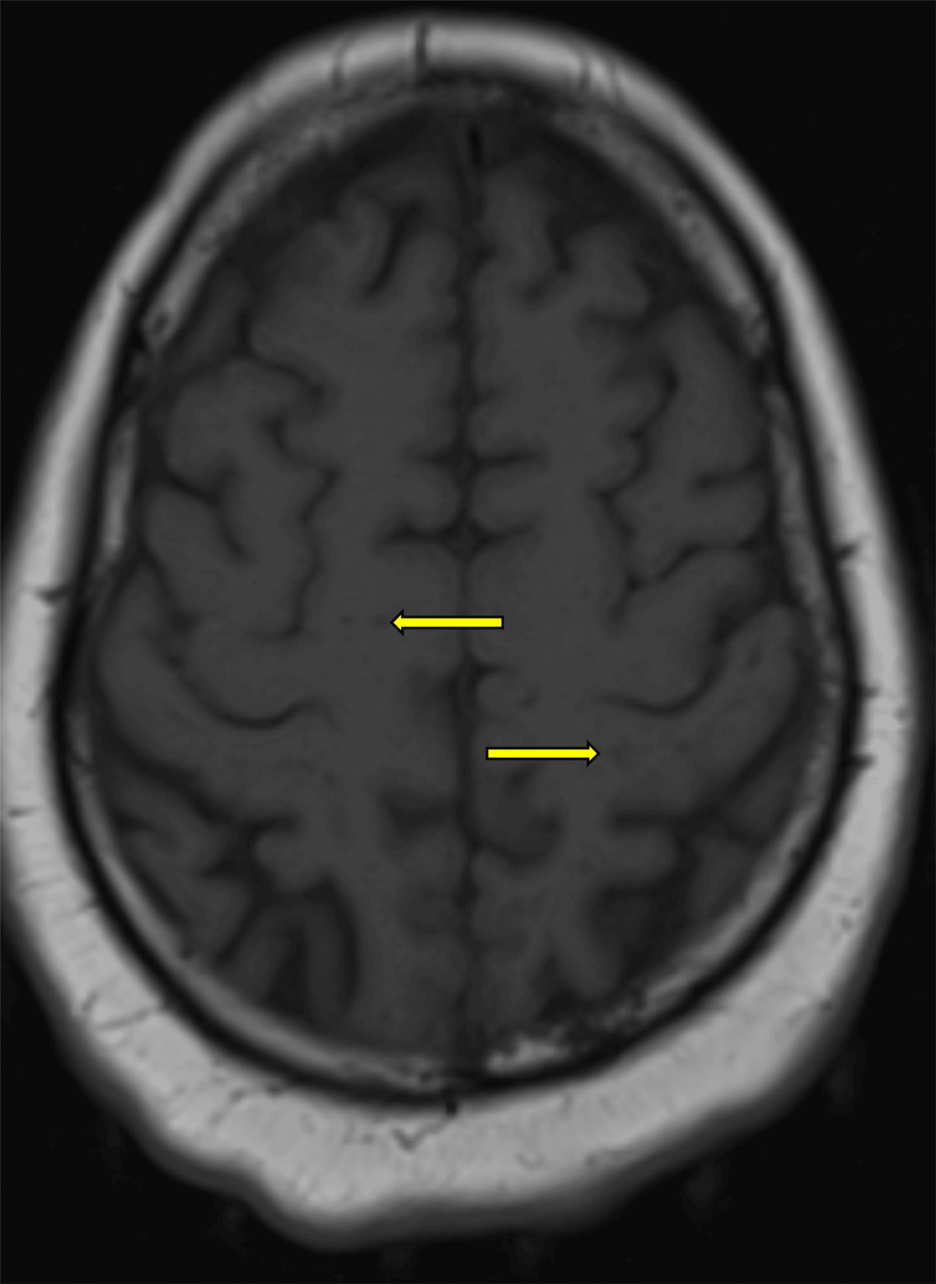
Multiple nodules (arrows) noted throughout the brain, consistent with tuberculosis granulomas in MRI.

After a two-week hospital stay, she self-discharged, flew back to the United Kingdom, and was readmitted. CT of the head revealed multiple tuberculous granulomas and a striato-capsular infarct. MRI of the brain confirmed CT findings (Figure 4). CT of the thorax revealed an aneurysm in the distal thoracic aorta (Figure 3).

Subsequently, her care was transferred to the infectious disease team at a tertiary care hospital urgently. She had a two-week admission there. An endovascular repair of the aortic aneurysm was undertaken, and antituberculous medication was continued. The third cranial palsy improved with regular eye appointments and correction glasses, and she was discharged from eye service in a year. Once she completed the 12-month antitubercular regime with regular infection disease follow-up, treatment was felt satisfactory and she was declared disease-free and discharged.

## Discussion

Our patient’s presentation lacked the typical pulmonary features of TB, instead initially manifesting with fever, malaise, and deranged liver function. These signs misled clinicians to suspect tropical or gastrointestinal infections, consistent with documented diagnostic delays in miliary TB due to its non-specific presentation [1,3]. A tree-in-bud appearance in chest X-ray (Figure 1) and widespread miliary nodules (Figure 2) present on imaging without the classical symptoms of cough, blood in sputum, or weight loss also supported the unpredictable presentation.

Neurological complications emerged later in the disease course, with isolated third cranial nerve palsy and a striato-capsular infarct confirmed via imaging. While CNS involvement is common in disseminated TB, isolated third nerve palsy is an uncommon presentation. Sixth nerve palsy is more frequently observed [4]. Cerebral infarction in TBM results from vasculitis and thrombosis due to basal exudates encasing cerebral vessels, especially in the circle of Willis, and is a known complication of advanced TB meningitis [5,8].

The co-occurrence of a large thoracic aortic aneurysm (Figure 2) in this patient added another layer of complexity. Tuberculous aortic aneurysms are rare, with direct spread from adjacent lymph nodes or paravertebral abscesses being the most common etiology [6,7]. Most cases are saccular pseudoaneurysms, and management typically requires endovascular or open surgical repair along with long-term antituberculous therapy [7]. Delayed diagnosis increases the risk of rupture and mortality.

Additionally, the evolution of symptoms during antituberculous treatment raises the possibility of paradoxical immune reconstitution inflammatory syndrome (IRIS), where clinical worsening occurs due to an exaggerated immune response. Though most commonly seen in HIV-infected individuals, IRIS is also reported in HIV-negative patients undergoing treatment for extrapulmonary TB and may require corticosteroids for management [9].

This case strongly emphasizes the need for a high index of suspicion for disseminated TB in patients presenting with unusual or multifocal symptoms, even in the absence of classic pulmonary signs. Comprehensive imaging, including brain and thoracic evaluations, was invaluable in establishing the diagnosis in this patient.

## Conclusions

TB can present in diverse ways, often mimicking other conditions. Therefore, maintaining a high index of suspicion is crucial for accurate diagnosis, as it is a curable disease when promptly diagnosed. Early detection and treatment are key to minimizing the morbidity associated with TB. Despite significant medical advancements, TB remains a deadly disease due to delayed diagnosis caused by misleading symptoms and signs. This case, with its rare combination of an isolated third cranial nerve palsy, multiple intracranial tuberculomas, and a thoracic aortic aneurysm in miliary TB, highlights the need for vigilance and a multidisciplinary approach to ensure timely diagnosis and management, ultimately improving patient outcomes.
